# Dual Solutions for Nonlinear Flow Using Lie Group Analysis

**DOI:** 10.1371/journal.pone.0142732

**Published:** 2015-11-17

**Authors:** Muhammad Awais, Tasawar Hayat, Sania Irum, Salman Saleem

**Affiliations:** 1 Department of Mathematics, COMSATS Institute of Information Technology, Attock, 43600, Pakistan; 2 Department of Mathematics, Quaid-I-Azam University 45320, Islamabad, 44000, Pakistan; Abdul Wali Khan University Mardan, PAKISTAN

## Abstract

`The aim of this analysis is to investigate the existence of the dual solutions for magnetohydrodynamic (MHD) flow of an upper-convected Maxwell (UCM) fluid over a porous shrinking wall. We have employed the Lie group analysis for the simplification of the nonlinear differential system and computed the absolute invariants explicitly. An efficient numerical technique namely the shooting method has been employed for the constructions of solutions. Dual solutions are computed for velocity profile of an upper-convected Maxwell (UCM) fluid flow. Plots reflecting the impact of dual solutions for the variations of Deborah number, Hartman number, wall mass transfer are presented and analyzed. Streamlines are also plotted for the wall mass transfer effects when suction and blowing situations are considered.

## Introduction

The non-Newtonian fluids at present are acknowledged more appropriate for scientific and technological applications than the materials obeying the Newtonian law of viscosity. However, there exists no single constitutive equation by which all the non-Newtonian materials can be described. Researchers and applied mathematicians have proposed a variety of constitutive equations in view of their rheological properties describing the non-Newtonian behavior. Such constitutive equations give rise to several complexities in the momentum equations which arises various challenges for researchers to model the problems in mathematical form and to evaluate/analyze the computed solutions mathematically and physically. The classifications of non-Newtonian fluids in general are classified into three types known as the differential, rate and integral. Available information on the topic indicates that much attention of the researchers has been accorded to the flows of second, third and fourth order fluids i.e. the subclasses of differential type materials. Undoubtedly these subclasses predicts the normal stress, shear thickening and thinning features but cannot describe relaxation time effect which many polymers show at low molecular weight. The relaxation time effect can be examined using Maxwell model (the simplest subclass of rate type fluid). The literature for flows of Maxwell fluid is quite sizeable. Few recent studies and several references on rheology of Maxwell fluid can be seen in the investigations [1–15].

As per our knowledge no attempt has been made for the Lie group analysis and the development of dual solutions for magnetohydrodynamic (MHD) [16–21] flow of Maxwell fluid over a permeable shrinking surface. Numerical solutions are presented for different cases of wall mass transfer. Graphical results are presented for the variations of different sundry parameters.

## Mathematical statement

Let us consider an upper-convected Maxwell (UCM) fluid flowing over a permeable shrinking sheet in a two-dimensional steady and incompressible field. The uniform magnetic field of strength *B*_0_ is applied along the $\bar{y}$—axis whereas the induced magnetic field is neglected due to the assumption of very small magnetic Reynold number whereas an upper-convected Maxwell (UCM) fluid occupies the region $\bar{y}≻0.$ The laws of conservations of mass and momentum for MHD boundary layer flow of an upper-convected Maxwell (UCM) fluid over a permeable shrinking surface are given by

$$\frac{\partial \bar{u}}{\partial \bar{x}}+\frac{\partial \bar{v}}{\partial \bar{y}}=0,$$ (1)

$$\bar{u}\frac{\partial \bar{u}}{\partial \bar{x}}+\bar{v}\frac{\partial \bar{u}}{\partial \bar{y}}+\lambda \left({\bar{u}}^{2}\frac{\partial^{2}\bar{u}}{\partial {\bar{x}}^{2}}+{\bar{v}}^{2}\frac{\partial^{2}\bar{u}}{\partial {\bar{y}}^{2}}+2\bar{u}\bar{v}\frac{\partial^{2}\bar{u}}{\partial \bar{x}\partial \bar{y}}\right)=\nu \frac{\partial^{2}\bar{u}}{\partial {\bar{y}}^{2}}-\frac{\sigma B_{0}^{2}}{\rho }(\bar{u}+\lambda \;\bar{v}\frac{\partial \bar{u}}{\partial \bar{y}}),$$ (2)

along with the boundary conditions

u¯=U¯w(x¯)=−C1x¯,v¯=v¯w(x¯),aty¯=0,u¯→0,asy¯→∞, (3)

in which $\bar{u}$ and $\bar{v}$ are the velocity components along $\bar{x}$ and $\bar{y}$ directions respectively, *ρ* represented the density, *ν* the kinematic viscosity, *σ* the electrical conductivity and ${\bar{U}}_{w}(\bar{x})$ the shrinking velocity with *C*_1_ > 0 is shrinking constant.

By using the following dimensionless quantities

$$x=\frac{C_{1}\bar{x}}{U_{1}},\;y=\sqrt{\frac{C_{1}}{\nu }}\bar{y},\;u=\frac{\bar{u}}{U_{1}},\;v=\frac{\bar{v}}{\sqrt{C_{1}\nu }},$$ (4)

Eqs (1–3) take the following forms

$$\frac{\partial u}{\partial x}+\frac{\partial v}{\partial y}=0,$$ (5)

$$u\frac{\partial u}{\partial x}+v\frac{\partial u}{\partial y}+\beta \left(u^{2}\frac{\partial^{2}u}{\partial x^{2}}+v^{2}\frac{\partial^{2}u}{\partial y^{2}}+2uv\frac{\partial^{2}u}{\partial x\partial y}\right)=\frac{\partial^{2}u}{\partial y^{2}}-M^{2}\left(u+\beta v\frac{\partial u}{\partial y}\right),$$ (6)

$$\begin{array}{l} \;u=-x,\;v=\frac{v_{w}}{\sqrt{C_{1}\nu }},\;\text{at}\;y=0,\\ u\to 0,\;\text{as}\;y\to \infty , \end{array}$$ (7)

where the Hartman number *M* and the Deborah number *β* are defined as follows:

$$M^{2}=\frac{\sigma B_{0}^{2}}{\rho C_{1}},\;\beta =\lambda C_{1}.$$ (8)

The stream function *ψ*(*x*, *y*) is introduced such as

$$u=\frac{\partial \psi }{\partial y},\;v=-\frac{\partial \psi }{\partial x},$$ (9)

Making use of Eq (9), law of conservation of mass (Eq (5)) is identically satisfied whereas Eqs (6) and (7) become

$$\psi_{y}\psi_{xy}-\psi_{x}\psi_{yy}+\beta \left(\psi_{y}^{2}\psi_{xxy}+\psi_{x}^{2}\psi_{yyy}-2\psi_{x}\psi_{y}\psi_{xyy}\right)=\psi_{yyy}-M^{2}\left(\psi_{y}-\beta \psi_{x}\psi_{yy}\right),$$ (10)

$$\begin{array}{l} \;\psi_{y}=-x,\;\psi_{x}=-\frac{v_{w}}{\sqrt{C_{1}\nu }},\;\text{at}\;y=0,\\ \psi_{y}\to 0,\;\text{as}\;y\to \infty . \end{array}$$ (11)

## Scaling group of transformation

In order to proceed for the Lie group analysis we consider

$$\Gamma =\{\begin{array}{l} x^{*}=xe^{\varepsilon \alpha_{1}},\;y^{*}=ye^{\varepsilon \alpha_{2}},\;\psi^{*}=\psi e^{\varepsilon \alpha_{3}},\\ \;u^{*}=ue^{\varepsilon \alpha_{4}},\;v^{*}=ve^{\varepsilon \alpha_{5}} \end{array}$$ (12)

Making use of Eq (12), the coordinates (*x*,*y*,*ψ*,*u*,*v*,*θ*,*ϕ*) transform into the coordinates (*x* ^*^, *y* ^*^, *ψ* ^*^, *u* ^*^, *v* ^*^, *θ* ^*^, *ϕ* ^*^) whereas Eqs (10 and 11) take the forms

$$\begin{array}{l} e^{\varepsilon (-2\alpha_{3}+2\alpha_{2}+\alpha_{1})}(\psi_{y^{*}}^{*}\psi_{x^{*}y^{*}}^{*}-\psi_{x^{*}}^{*}\psi_{y^{*}y^{*}}^{*})-e^{\varepsilon (-\alpha_{3}+3\alpha_{2})}\psi_{y^{*}y^{*}y^{*}}^{*}\\ +\beta e^{\varepsilon (-3\alpha_{3}+3\alpha_{2}+2\alpha_{1})}\psi_{y^{*}}^{{*}^{2}}\psi_{x^{*}y^{*}x^{*}}^{*}+\psi_{x^{*}}^{{*}^{2}}\psi_{y^{*}y^{*}y^{*}}^{*}-2\psi_{x^{*}}^{*}\psi_{y^{*}}^{*}\psi_{x^{*}y^{*}y^{*}}^{*}\\ \;+M^{2}\left(e^{\varepsilon (-\alpha_{3}+\alpha_{2})}\psi_{y^{*}}^{*}+e^{\varepsilon (-2\alpha_{3}+2\alpha_{2}+\alpha_{1})}\beta \psi_{x^{*}}^{*}\psi_{y^{*}y^{*}}^{*}\right)=0, \end{array}$$ (13)

$$\begin{array}{l} \;e^{\varepsilon (-\alpha_{3}+\alpha_{2})}\psi_{y^{*}}^{*}=-e^{-\varepsilon \alpha_{1}}x^{*},\;e^{\varepsilon (-\alpha_{3}+\alpha_{1})}\psi_{x^{*}}^{*}=-\frac{v_{w}}{\sqrt{C_{1}\nu }},\;\text{at}\;y^{*}=0,\\ e^{\varepsilon (-\alpha_{3}+\alpha_{2})}\psi_{y^{*}}^{*}\to 0,\;\text{when}\;y^{*}\to \infty . \end{array}$$ (14)

It is noted that the system remains invariant under the group of transformations Γ and we get the following relation among parameters

$$\begin{array}{l} -2\alpha_{3}+2\alpha_{2}+\alpha_{1}=-3\alpha_{3}+3\alpha_{2}+2\alpha_{1}=-\alpha_{3}+3\alpha_{2}=-\alpha_{3}+\alpha_{2},\\ \;-\alpha_{3}+\alpha_{1}=0,\\ \;-\alpha_{3}+\alpha_{2}=-\alpha_{1}. \end{array}$$ (15)

By solving the system given in Eq (15) we obtain

$$\alpha_{1}=\alpha_{3}=\alpha_{4}\;\text{and}\;\alpha_{2}=\alpha_{5}=0.$$ (16)

Making use of Eq (16), the set of transformations Γ reduces to

$$\Gamma =\{\begin{array}{l} x^{*}=xe^{\varepsilon \alpha_{1}},\;y^{*}=y,\;\psi^{*}=\psi e^{\varepsilon \alpha_{1}},\\ \;u^{*}=ue^{\varepsilon \alpha_{1}},\;v^{*}=v. \end{array}$$ (17)

Expanding by Taylor's method in powers of *ε* and retaining terms up to the order *ε*, we get

$$\Gamma =\{\begin{array}{l} x^{*}-x=x\varepsilon \alpha_{1},\;\psi^{*}-\psi =\psi \varepsilon \alpha_{1},\\ \;u^{*}-u=u\varepsilon \alpha_{1},\;v^{*}-v=y^{*}-y=0. \end{array}$$ (18)

In terms of differentials we get

$$\frac{dx}{\alpha_{1}x}=\frac{dy}{0}=\frac{d\psi }{\alpha_{1}\psi }=\frac{du}{\alpha_{1}u}=\frac{dv}{0}.$$ (19)

Solving the above equations we acquire

$$y^{*}=\eta ,\;\psi^{*}=x^{*}f(\eta ).$$ (20)

It is noted that with the help of the above relations, Eq (13) becomes

$$f^{\prime \prime \prime }+ff^{\prime \prime }-f^{\prime 2}-\beta (f^{2}f^{\prime \prime \prime }-2ff^{\prime }f^{\prime \prime })-M^{2}(f^{\prime }+\beta ff^{\prime \prime })=0,$$ (21)

along with the boundary conditions

$$\begin{array}{l} \;f=S,\;f^{\prime }=-1,\;\text{at}\;\eta =0,\\ f^{\prime }\to 0\;\text{as}\;\eta \to \infty . \end{array}$$ (22)

## Solution methodology

### Shooting method:

The differential Eq (21) along with conditions (22) is solved numerically using an efficient approach namely shooting method. Runge-Kutta fourth-order algorithm combined with Newtons' method is utilized to approximate the shoot values in order to match at a finite value of η → *∞* say η_*∞*_. For this we first write

$$f^{\prime }=f_{1},\;f_{1}^{\prime }=f_{2},\;f_{2}^{\prime }=\frac{f_{1}^{2}-\left(M^{2}\beta +1\right)\;ff_{2}-2\beta ff_{1}f_{2}+M^{2}f_{1}}{1-\beta f^{2}},$$

with conditions

$$\begin{array}{l} f(0)=S,\;f_{1}(0)=-1,\\ f_{2}(0)=? \end{array}$$

It is noted that in order to compute the solution of the above differential equation as an initial value problem, we require the value of *f*_2_(0), whereas no such value is given initially. In order to compute the desired result we select an initial guess and then applied the fourth-order Runge-Kutta method to approximate the value up to the desired accuracy of 10^−5^.

## Results and discussion

**Fig 1:**
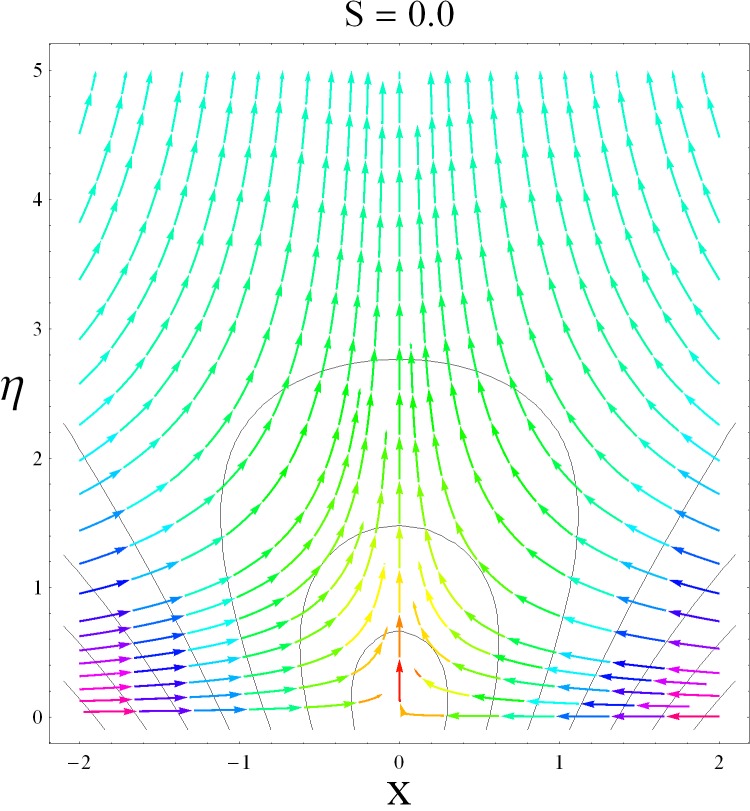
Streamlines without suction/injection.

**Fig 2:**
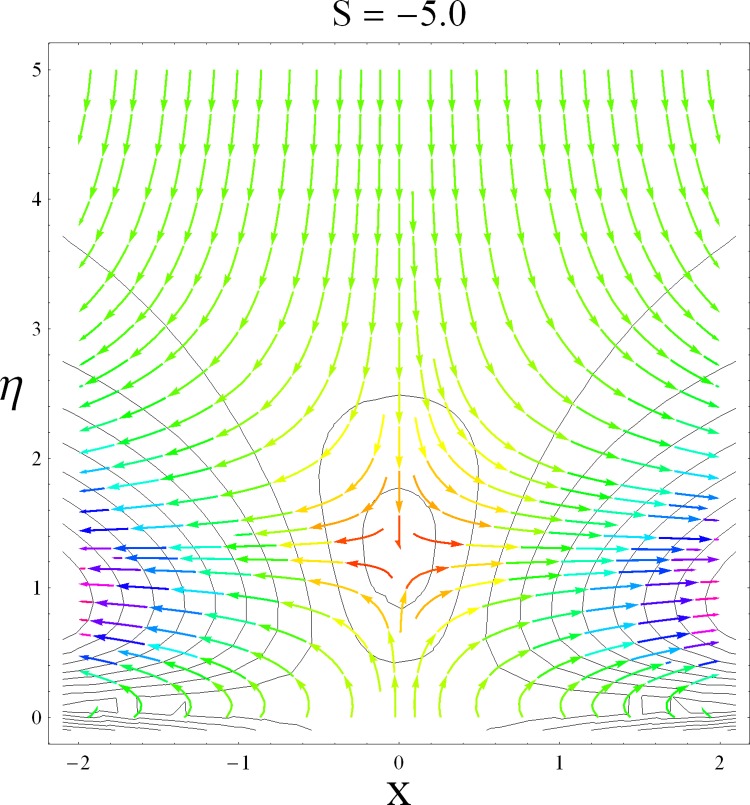
Stream lines for injection case.

**Fig 3:**
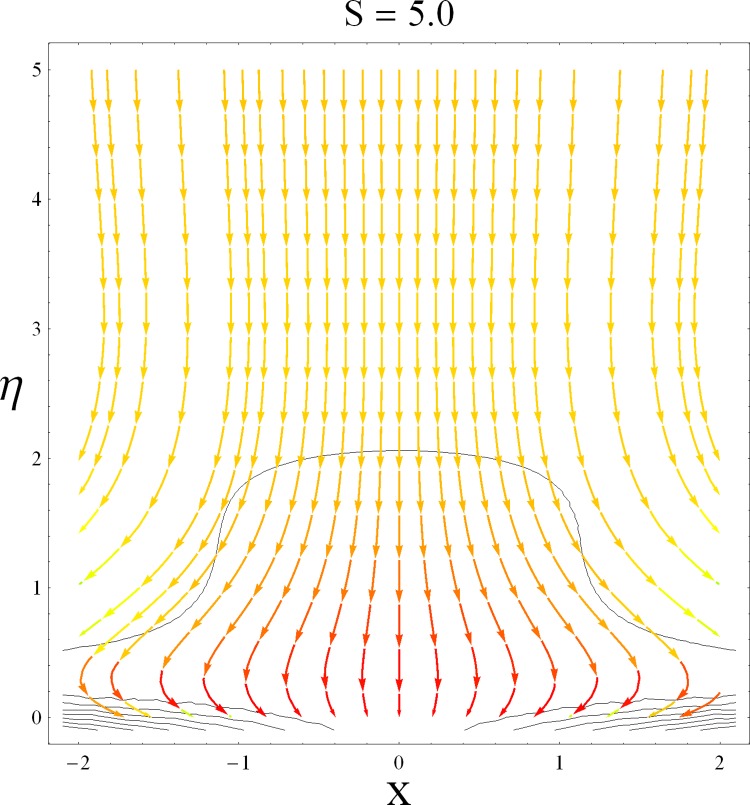
Streamlines for suction case.

**Fig 4:**
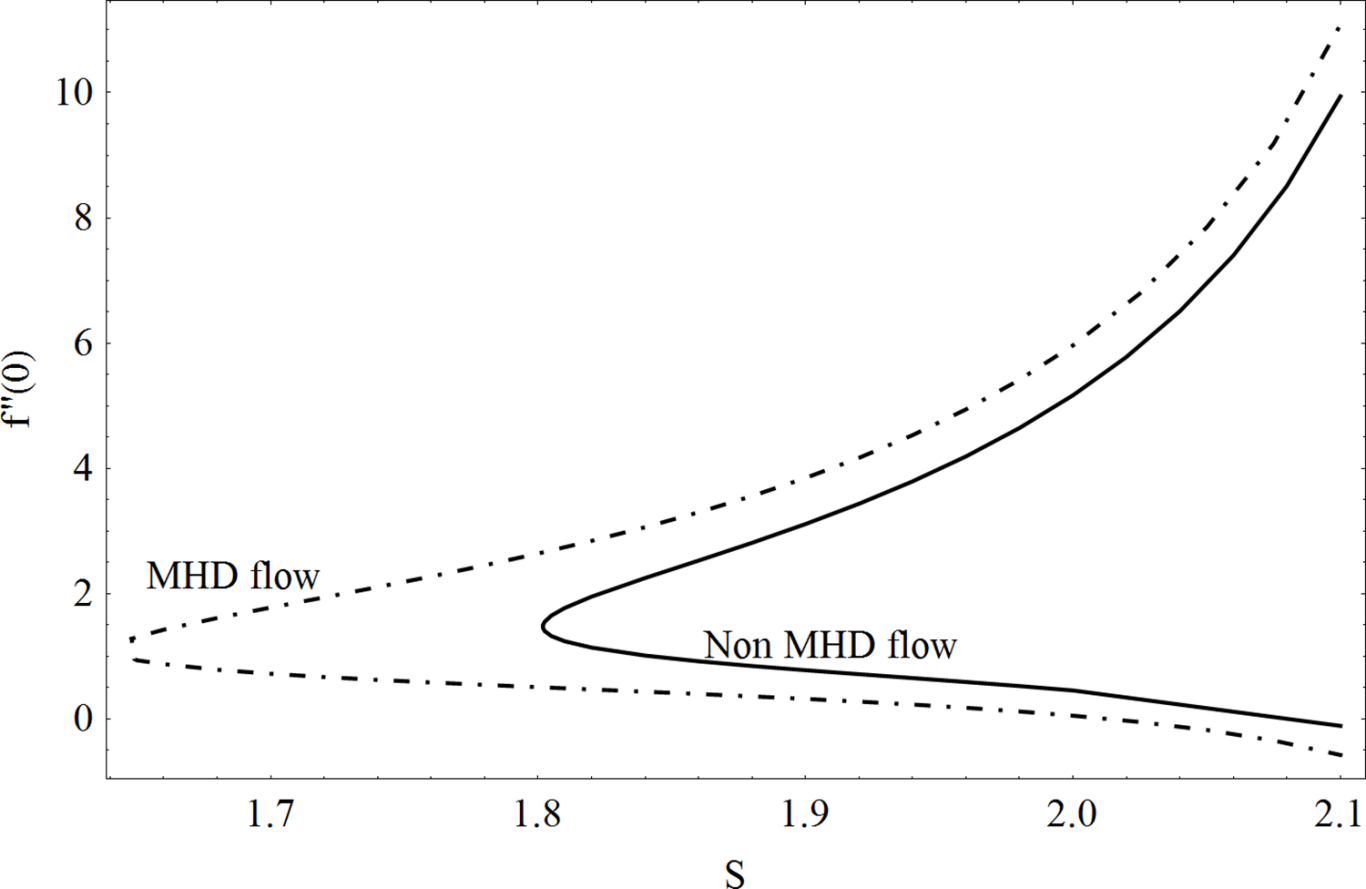
Dual solutions: MHD Vs hydrodynamic case.

**Fig 5:**
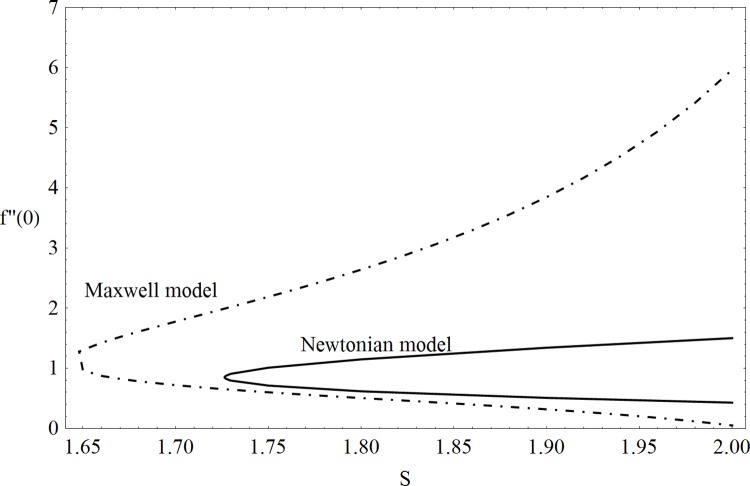
Dual solutions: Newtonian vs Maxwell model.

**Fig 6:**
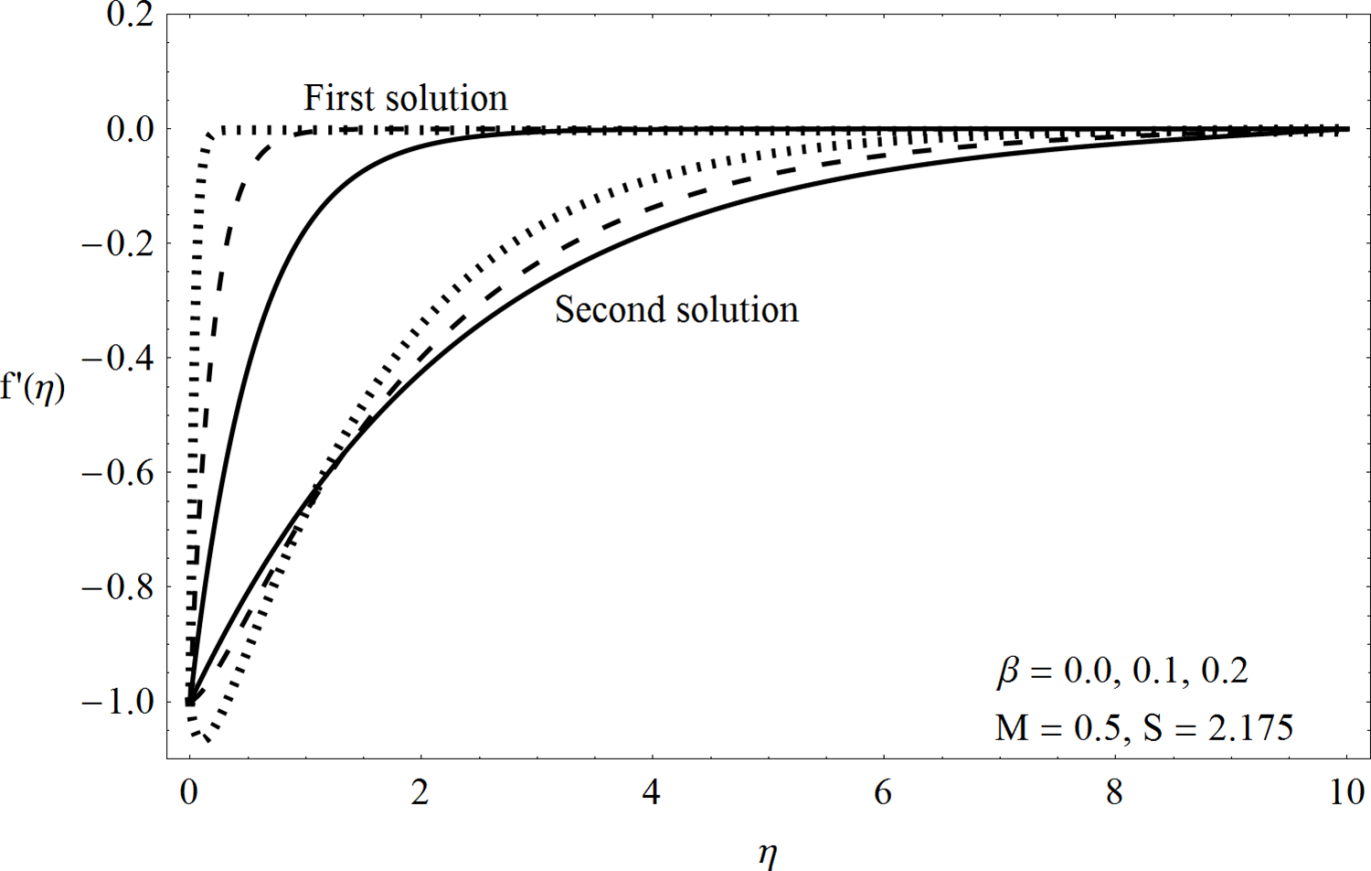
Dual solutions (*f*′(*η*)) for Deborah number.

**Fig 7:**
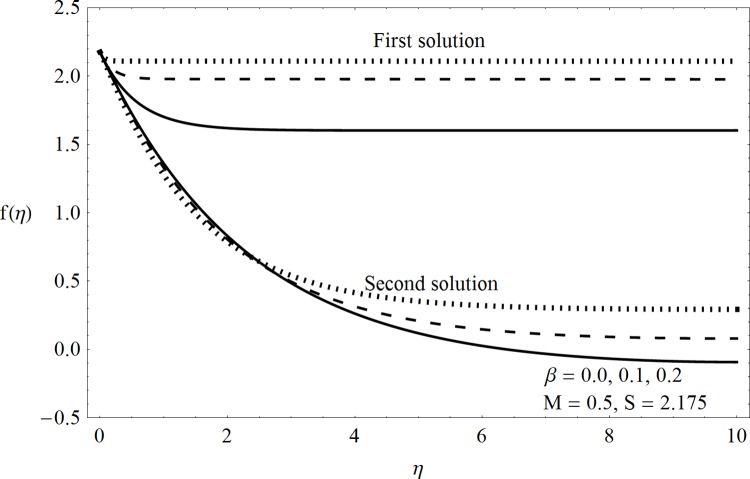
Dual solutions (*f*(*η*)) for Deborah number.

**Fig 8:**
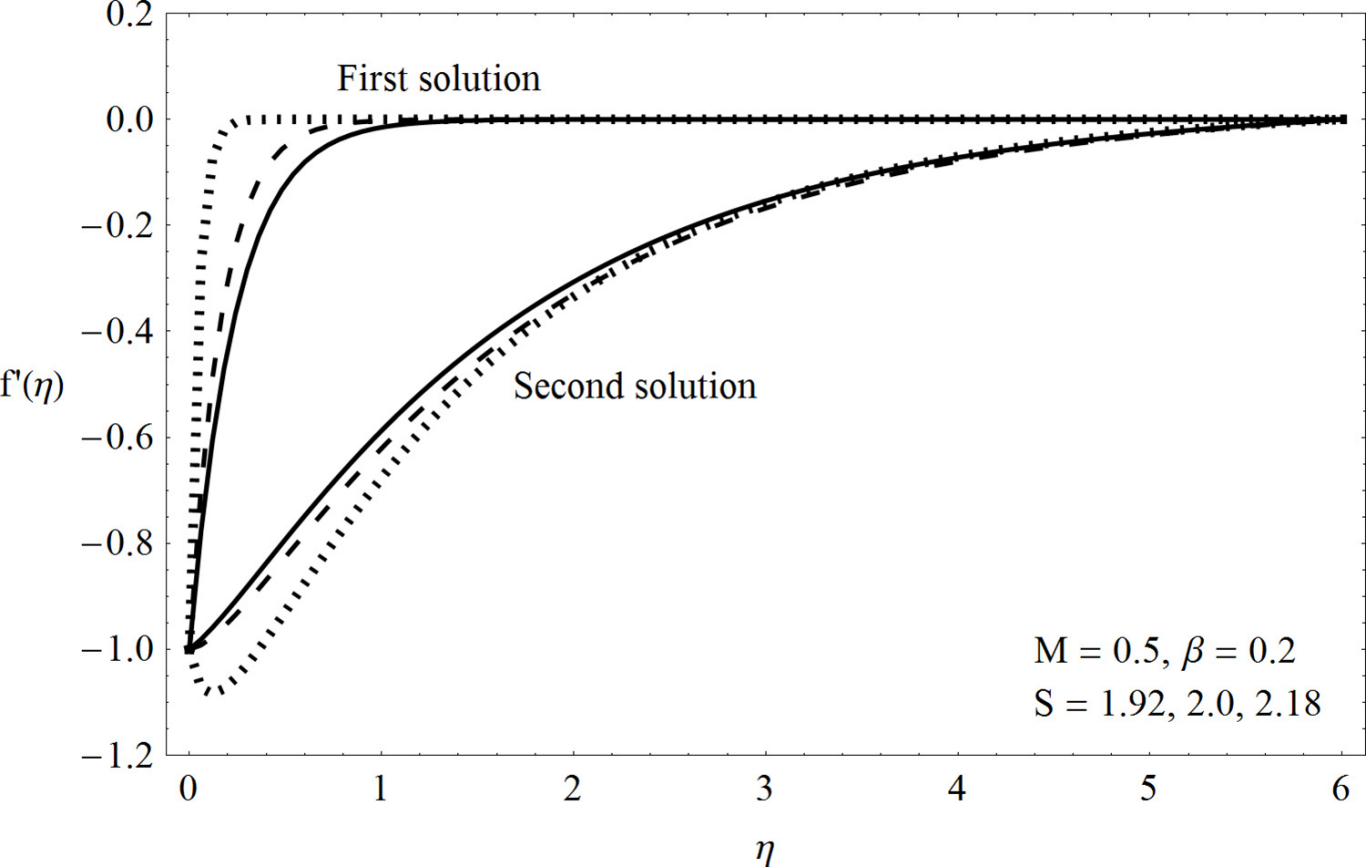
Dual solutions (*f*′(*η*)) for suction.

**Fig 9:**
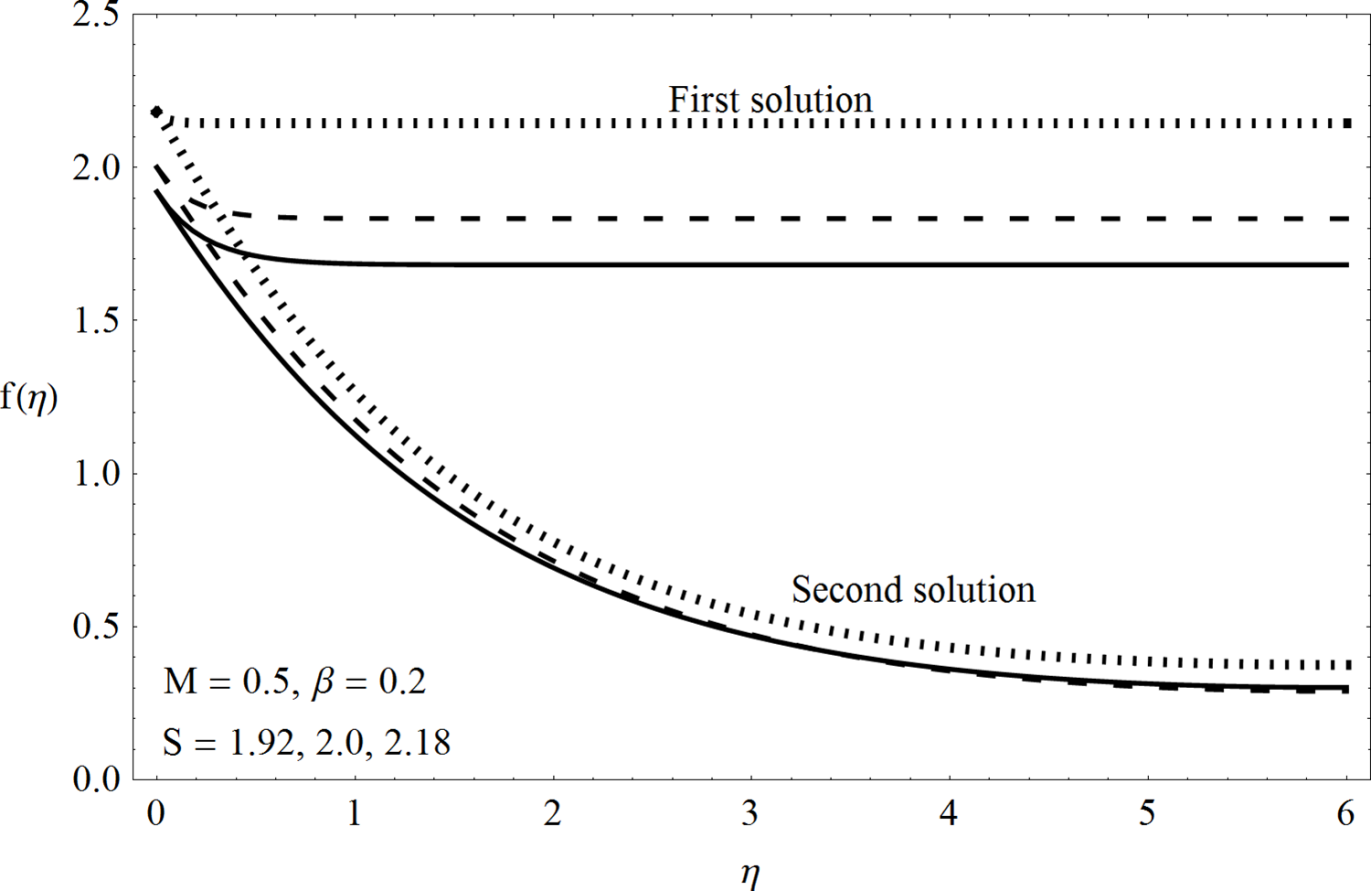
Dual solutions (*f*(*η*)) for suction.

**Fig 10:**
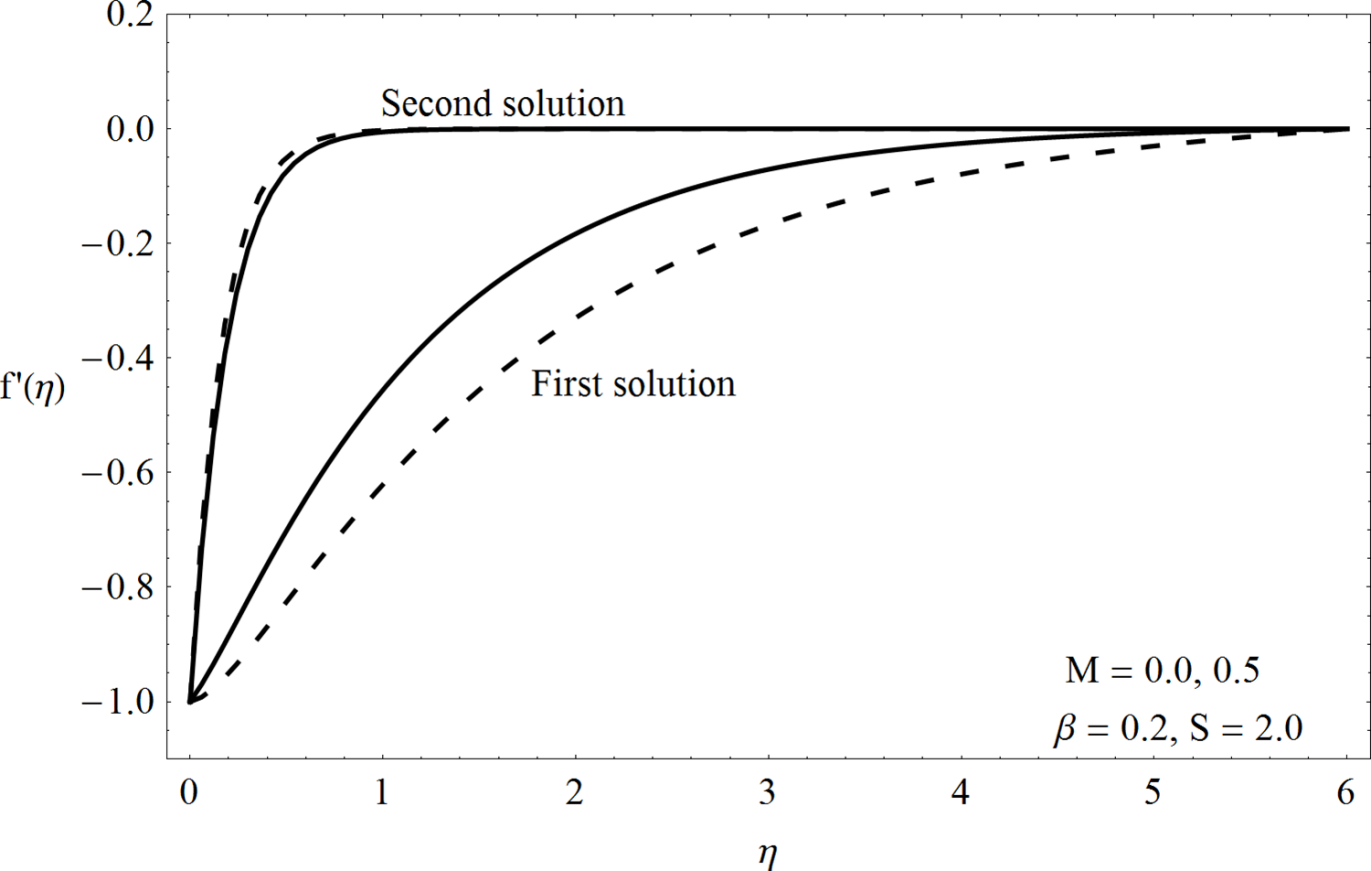
Dual solutions (*f*′(*η*)) for MHD case.

**Fig 11:**
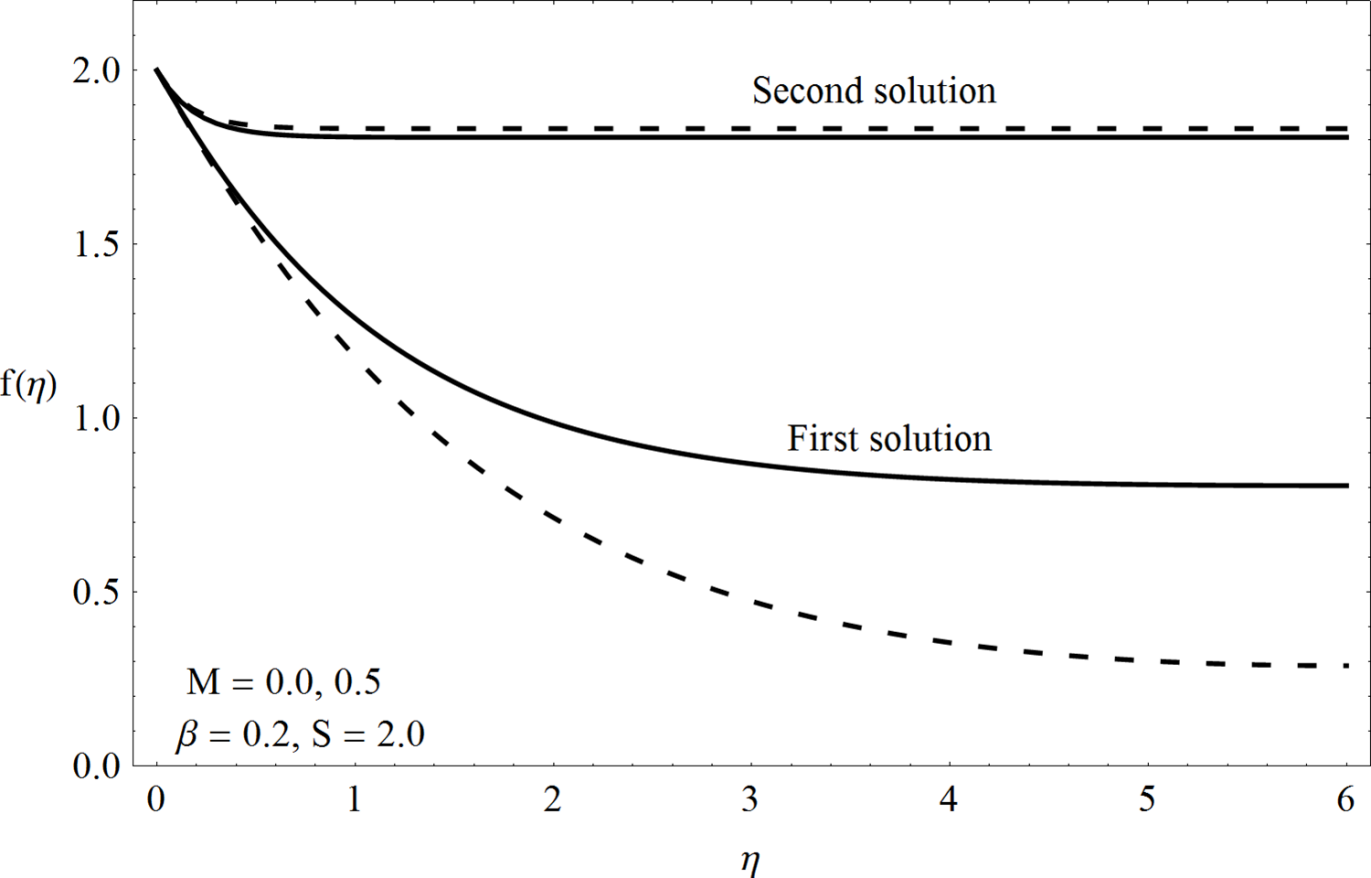
Dual solutions (*f*(*η*)) for MHD case.

This part of the paper corresponds to some graphical observations so the one can analyze the physical insight of the problem. Figs 1–3 are prepared to show the streamlines for different cases of suction and injection. It is observed from Fig 1 that when the suction/injection phenomenon is absent then the viscous boundary layer is very much thinner and the flow near the shrinking wall is dominant whereas injection at the wall adds up an extra force which causes the boundary layer to become thicker as shown in Fig 2. Fig 3 presents the streamlines for the suction case which are found to be qualitatively opposite when compared to the injection case. Larger suction in combination with the shrinking acts as a supporting agent to suction the fluid out of the system. Fig 4 portrays the dual nature of solutions for the magnetohydrodynamic (MHD) vs hydrodynamic cases. It is observed that for hydrodynamic case, the two-dimensional flow of upper-convected Maxwell (UCM) fluid over a permeable wall is possible only when the wall mass transfer satisfies the inequality *S* ≥ 1.8018 (see Fig 4). Moreover it is elucidated from this plot that for the magnetohydrodynamic case, the wall mass suction inequality reduces to the value *S* ≥ 1.648 due to the decrease in the intrinsic fluidity of the material. Fig 5 presents the difference between the flow of Newtonian and Maxwell fluids for some values of suction parameter S. It is observed that the dual nature of solution for Newtonian fluid model (*β* = 0.0) exists only when the wall mass transfer satisfies the inequality *S* ≥ 1.7264 whereas for Maxwell fluid model (*β* > 0.0) the inequality reduces to *S* ≥ 1.6. Moreover dual nature of the solutions for both hydrodynamic and hydromagnetic flow situations are presented and found quite different. It is observed that the value of skin friction increase rapidly for the first solution whereas it decreases for the second solution but the variations in first solution is quite significant when compared with the other. Physically we can say that the increase in Deborah number *β* results into a decrease in a vorticity generation of a Maxwell fluid. Since the vorticity generation due to shrinking for Maxwell fluid is not as much as of Newtonian fluid therefore the boundary layer separation for Maxwell fluid is delayed and the similar solution exists for lesser amount of wall mass transfer. Moreover it is also noted that for the Newtonian fluid flow, both solutions are closed to each other and nearly parallel to x-axis which shows the linear behavior of the skin friction whereas for the non-Newtonian fluid, the variations in both solutions are quite significant and found quite opposite. Figs 6 and 7 present the effects of Deborah number *β* on the velocity fields *f*′ and *f* respectively. It is observed that the dual solutions for velocity against different values of *β* exist. It is observed that the non-Newtonian fluid behaves much like liquids (such as paints, polymer solutions etc.) for smaller value of Deborah number however for larger values of Deborah number, fluid transforms from liquids to the position of becoming a viscoelastic solid (such as toothpaste, jelly or rubber etc.). Thus the flow retards by increasing Deborah number which is quite in agreement with the Fig 6. It is also elucidated from Figs 6 and 7 that velocity increases with an increase in Deborah number *β* for the first solution whereas from second solution, the oscillatory effects and cross over is found. The effects of wall mass suction (due to the permeability of the surface) on the flow are demonstrated in the Figs 8 and 9. The dual solutions exist for different values of *S* and both solutions show quite opposite behavior. The magnitude of first solution enhances with an increasing in the suction rate whereas the magnitude of second solution decreases. Figs 10 and 11 present the influence of magnetic field *M* on the velocity. It is observed that dual nature of solutions exists for different values of *M*. It is also illustrated that the first solution is dominant in comparison to the second solution (see Figs 10 and 11). It is perceived that when any fluid is subjected to a magnetic field then its apparent viscosity increases to the point of becoming a viscoelastic solid. Importantly, the velocity and the rheology of the fluid can be controlled very accurately by varying the magnetic field intensity. The outcome of which is that the fluid's ability to transmit force can be controlled with the help of an electromagnet which gives rise to its many possible control-based applications including MHD power generation, electromagnetic casting of metals, MHD ion propulsion etc. Moreover nomenclature have been identified in Table 1.

**Table 1:** Nomenclature

*B*_0_	applied magnetic field	*Greeks symbol*	
$\bar{u},$ $\bar{v}$	velocity components	*λ*	convection parameter
$\bar{x},$ $\bar{y}$	Cartesian coordinates	*ψ*	stream function
*U*_*w*_	shrinking velocity	*Β*	Deborah number
*V*_*w*_	porosity of wall	*ρ*	density
*M*	Hartman number	Γ	scaling set
*C*_1_	dimensional constant	*η*	similarity variable
*f*	dimensionless stream function	*σ*	electrical conductivity
*S*	suction/blowing	*ν*	kinematical viscosity

## Final outcomes

The main results of the present analysis are mentioned below:

- Larger values of Deborah number β results in to steady flow of non-Newtonian fluid with lessor amount of wall mass transfer.
- Momentum boundary layer decreases with Deborah number in both first and second solutions.
- Wall mass transfer has opposite effects on the viscous boundary layer in first and second solutions.
- Magnetic field retards the rheology and the dynamics of the flow field.
- Suction and blowing phenomena show the opposite behavior on the streamlines.
